# Giant Extensional Strain of Magnetoactive Elastomeric Cylinders in Uniform Magnetic Fields

**DOI:** 10.3390/ma13153297

**Published:** 2020-07-24

**Authors:** Dmitry V. Saveliev, Inna A. Belyaeva, Dmitry V. Chashin, Leonid Y. Fetisov, Dirk Romeis, Wolfgang Kettl, Elena Yu. Kramarenko, Marina Saphiannikova, Gennady V. Stepanov, Mikhail Shamonin

**Affiliations:** 1 Research and Education Center “Magnetoelectric Materials and Devices”, MIREA - Russian Technological University, 119454 Moscow, Russia; dimsav94@gmail.com (D.V.S.); chashindv@yandex.ru (D.V.C.); fetisovl@yandex.ru (L.Y.F.); 2 East Bavarian Centre for Intelligent Materials (EBACIM), Ostbayerische Technische Hochschule (OTH) Regensburg, Seybothstr. 2, 93053 Regensburg, Germany; inna.belyaeva@oth-regensburg.de (I.A.B.); wolfgangkettl@rocketmail.com (W.K.); 3 Leibniz-Institut für Polymerforschnung Dresden e.V., 01069 Dresden, Germany; romeis@ipfdd.de (D.R.); grenzer@ipfdd.de (M.S.); 4 Faculty of Physics, Lomonosov Moscow State University, 119991 Moscow, Russia; kram@polly.phys.msu.ru (E.Y.K.); gstepanov@mail.ru (G.V.S.); 5 A.N. Nesmeyanov Institute of Organoelement Compounds of Russian Academy of Sciences, 119991 Moscow, Russia; 6 State Scientific Center of the Russian Federation, Institute of Chemistry and Technology of Organoelement Compounds, 111123 Moscow, Russia

**Keywords:** magnetostriction, magnetoactive elastomer, extensional strain, hysteresis, magnetomechanical effect, magnetodeformation

## Abstract

Elongations of magnetoactive elastomers (MAEs) under ascending–descending uniform magnetic fields were studied experimentally using a laboratory apparatus specifically designed to measure large extensional strains (up to 20%) in compliant MAEs. In the literature, such a phenomenon is usually denoted as giant magnetostriction. The synthesized cylindrical MAE samples were based on polydimethylsiloxane matrices filled with micrometer-sized particles of carbonyl iron. The impact of both the macroscopic shape factor of the samples and their magneto-mechanical characteristics were evaluated. For this purpose, the aspect ratio of the MAE cylindrical samples, the concentration of magnetic particles in MAEs and the effective shear modulus were systematically varied. It was shown that the magnetically induced elongation of MAE cylinders in the maximum magnetic field of about 400 kA/m, applied along the cylinder axis, grew with the increasing aspect ratio. The effect of the sample composition is discussed in terms of magnetic filler rearrangements in magnetic fields and the observed experimental tendencies are rationalized by simple theoretical estimates. The obtained results can be used for the design of new smart materials with magnetic-field-controlled deformation properties, e.g., for soft robotics.

## 1. Introduction

Magnetoactive elastomers (MAEs) are promising materials for manufacturing magnetic-field controlled linear actuators [1,2,3,4,5], in particular for soft robotics [6]. They consist of micrometer-sized soft magnetic particles (usually, iron) embedded into a non-magnetic elastomer matrix [7,8,9,10,11,12,13,14,15,16]. The practical reason can be found in their large magnetodeformations (up to ≈10^−1^) in feasible homogeneous magnetic fields (above 300 mT), although the intrinsic magnetostriction (MS) of a ferromagnetic filler is significantly lower (≈10^−6^). Lanotte et al. [17] identified all the contributions to magnetoelasticity of magnetic particles within a silicone matrix. The magnetodeformation of MAEs in a uniform magnetic field is also often denoted in the literature as magnetostriction [15], although the origin of this effect in MAEs is different from its origin in traditional magnetostrictive materials (crystalline ferromagnets). The physical origin is not the magnetization–strain coupling caused by the quantum spin–orbit interaction in ferromagnetic inclusions but the restructuring (RS) of the filler, i.e., changes in mutual arrangement of magnetized micrometer-sized inclusions, caused by magnetic interactions and restricted by the matrix elasticity. Such a significant reconfiguration becomes possible when the matrix is sufficiently compliant (Young’s modulus < 100 kPa). In inhomogeneous magnetic fields [18], even larger deformations become possible; elongations of several hundred percent have been observed in MAE specimens [19,20,21]. Very recently, other magnetoelastic effects related to MS, the Villary effect [22,23] and the Wiedemann effect [24], have been reported in compliant (mechanically soft) MAE materials as well.

Hitherto, several basic methods are available to measure the deformation of conventional ferromagnetic samples subjected to external magnetic fields: strain gauges [25,26] and optical [27,28] and capacitive [29,30] methods. The use of a particular method of strain measurements depends on the expected magnitude of the magnetostrictive effect, the temperature range, and the shape and size of the specimens. Most often, measurements are carried out on samples in the form of plates or cylinders. Existing measurement methods work well for small magnitudes of magnetic deformation λ ~10^−6^–10^−3^, but they do not allow measuring strains of tens of percent that are observed in MAEs. In addition, classical magnetostrictive materials are usually quite rigid (the magnitude order of the elastic modulus is 10^11^ Pa), while MAE can be much softer (elastic modulus is of the order of 10^5^ Pa or less). For example, attachment of a conventional strain gauge to a soft MAE may alter its deformation [31]. Hitherto, there are no generally accepted methods for measuring large deformations of such soft materials.

Only few papers devoted to the measurement of MS in MAEs can be found in the literature. To the best of our knowledge, the first measurement of MS in MAEs was reported by Bednarek [32] more than 20 years ago. The relative elongation of about 0.01 was observed in magnetic fields up to 8 T. Besides, the magnetostriction hysteresis was found in this investigation. The explanation was given in terms of displacement and rotation of magnetized particles in external magnetic fields. Today, we would call it the restructuring of the filler. The capacitive method was used for the measurement of magnetostriction. A specifically designed capacitor was employed. Six years later, the same author investigated MS in porous MAEs [33], where the maximum MS of 0.0481 was measured. Three years after the pioneering paper by Bednarek, Ginder et al. [34] measured the MS of a preloaded MAE cylindrical sample using a lever arm equipped with low-friction sapphire bearings at its pivot point. At the free end of the lever, a linear variable differential transformer (LVDT) was affixed to monitor the deflection produced by length changes in the sample. The maximum measured strain was about 0.003. The relative elongation of soft MAEs reported by Abramchuk et al. [35] reached 0.16. Martin et al. [36] investigated structured MAEs, where the Fe or Ni particles have been aligned during crosslinking in external magnetic fields. The largest measured strain was about 0.01; a greater MS response in all fields of a sample with the higher aspect ratio was explained by the opposing effect of demagnetizing fields. The experimental setup used a sophisticated optical cantilever apparatus with an impressive displacement resolution of ~50 nm. Gollwitzer et al. [37] measured the deformation of a ferrogel ball using image processing. Guan et al. [38] investigated the MS in isotropic MAEs with three different volume fractions of iron particles and compared an isotropic MAE with two anisotropic samples with the same filler concentration but different orientations of the chain-like aggregates. The conclusion was that the MS increases with the growing concentration of filler particles and the orientation of chain-like aggregates influences the MS significantly. The observed effect was small; the largest MS was only 184 ppm. Because the particles were of irregular shape, the proposed mechanism was rotation of elongated particles in applied magnetic fields. The MS was measured by an unspecified strain gauge. Diguet et al. [39] studied the effect of the aspect ratio (length to the diameter) of cylindrical samples on the saturation MS. It was observed that the saturation elongation decreases with the increasing aspect ratio. They also suggested a simple model based on the demagnetizing field and the effective Young’s modulus of a composite material. Further, they predicted and experimentally determined the optimal value of the filling factor (volume fraction) φ≈0.27, where the saturation MS should be the highest. This value was measured for the flattest sample and was equal to 0.092 in the maximum field of 955 kA/m. The measurement system was based on the image analysis from a CCD camera. The elastomeric matrix had the Young’s modulus of 140 kPa. Assuming that the elastomer was practically incompressible (the Poisson’s ratio *ν* ≈ 0.5), the shear modulus of the matrix was approximately 47 kPa. In modern MAEs, the shear modulus of an elastomeric matrix can be much lower, e.g., below 10 kPa [15,16]. Later, the same authors studied the influence of temperature on the MS of isotropic MAEs and found that the MS decreases with the decreasing temperature that can be explained by the increase in the elastic modulus [40]. Gong et al. [31] used digital holographic interferometry to analyze the morphology transformation of a cylindrical MAE sample. It was shown that such a deformation is not homogeneous due to the heterogeneity of the material. Both contractive and stretching deformations were observed over the specimen surface.

Significant theoretical efforts have been spent to understand the underlying mechanism of MS in MAEs [18,41,42,43,44,45,46,47,48,49,50,51,52,53]. There is a general consensus that the resulting strain is a consequence of two competing physical effects: first, the interaction between the magnetization vector and the demagnetizing field on the macroscopic scale; and, second, the interaction of magnetized particles on the mesoscopic scale $∼{\left(N/V\right)}^{-1/3}$, where *N* is the number of particles in the sample and *V* is the volume of the sample [52]. Unfortunately, the theory did not reach the state yet, where fabrication guidelines can be provided in order to design a MAE specimen with given magnetostrictive properties. Therefore, experimental investigations of magnetostrictive phenomena in soft MAEs are of particular importance. Hitherto, the available experimental results are rather fragmentary and refer to the earlier generations of MAEs. In recent years, significant progress has been achieved in fabricating mechanically soft MAE materials with the shear modulus less than 100 kPa, so that larger magnetostrictive strains can be achieved in smaller magnetic fields. Unfortunately, the published experimental techniques are rather sophisticated and/or expensive. This limits their spreading in the engineering and scientific communities. 

The purpose of this paper is twofold: First, we present a simple experimental setup allowing one to measure the strain of MAE samples with the shear modulus as low as 30 kPa. Second, this setup is used to characterize a number of MAE samples with different rigidity and ferromagnetic filler content. The obtained results are discussed with respect to their validity and interpreted in terms of the restructuring of the filler. We emphasize that the proposed setup allows one to characterize cylindrical samples with the sizes in all three dimensions of the order of 10 mm. The possibility of working with such voluminous specimens is important for envisaged applications of MAEs as soft magnetic-field-controlled actuators, in particular for soft robotics [6].

This paper is organized as follows. In Section 2, we describe the fabrication of MAE cylinders and the experimental setup. Experimental results are presented and discussed in Section 3 using some theoretical rationalizations and estimates. The results are summarized in the concluding Section 4, where an outlook about further investigations and perspectives is also made. 

## 2. Materials and Methods 

We fabricated 15 cylindrical isotropic-MAE specimens: three different cylinders (denoted as short, medium, and tall) were obtained for five different material compositions. Three of them were manufactured with the same iron content $w_{\mathrm{Fe}}$ of 80 mass% (*φ *≈ 33 vol%) and each having effective shear storage moduli in the absence of a magnetic field G_0_ (about 30, 50, and 120 kPa). The two other materials had the effective shear storage modulus in the absence of a magnetic field of about 30 kPa, but different iron content of 70 mass% (*φ * ≈ 22 vol%) and 75 mass% (*φ * ≈ 27 vol%). The synthesis of MAE materials followed the principles described by us in [54,55]. A short description is provided below.

The base polymer VS 100,000 (vinyl-functional polydimethylsiloxane) for addition-curing silicones, the chain extender modifier 715 (SiH-terminated polydimethylsiloxane), the reactive diluent polymer MV 2000 (monovinyl functional polydimethylsiloxane), the crosslinker 210 (dimethylsiloxane-methyl hydrogen siloxane copolymer), the Pt-Catalyst 510, and the Inhibitor DVS were provided by Evonik Hanse GmbH, Geesthacht, Germany. The silicone oil WACKER^®^ AK 10 (linear, non-reactive polydimethylsiloxane) was purchased from Wacker Chemie AG, Burghausen, Germany. The soft-magnetic carbonyl iron powder (CIP) type SQ (mean particle size d50 of 4.5 μm, no coating), provided by BASF SE Carbonyl Iron Powder & Metal Systems (Ludwigshafen, Germany), was used as the ferromagnetic filler. Magnetic properties of CIPs and MAEs with similar concentrations of iron particles can be found in [56,57,58]. The X-ray diffraction (XRD) analysis of CIPs was reported, e.g., in [59,60,61]. The phase analysis of different types of CIP did not show any visible difference in their composition [59]. The polymer VS 100,000, the polymer MV 2000, the modifier 715, and the silicone oil AK 10 were put together and blended with an electric mixer (Roti^®^-Speed-stirrer, Carl Roth GmbH, Germany) to form an initial compound.

In the next step, the initial compound was mixed with CIP and crosslinker 210. The crosslinking (hydrosilylation) reaction was activated by the Pt-Catalyst 510. For the activity control of the Pt-Catalyst, the inhibitor DVS was employed.

The specimens are classified in the following manner: the first number denotes the mass fraction of carbonyl iron in the composite material $w_{\mathrm{Fe}}$, the second number stands for the low-frequency shear storage modulus of the sample G_0_, and the last letter designates the height of the sample (Short/Medium/Tall). All samples have approximately the same diameter *d* of 14.8 mm. The aspect ratio is the ratio of the sample height *h*_0_ to the diameter: $\gamma =h_{0}/d$. For example, 80-50-T means the tall sample with 80 mass% of carbonyl iron and the shear modulus of about 50 kPa (maximum aspect ratio among all samples from this material).

Table 1 summarizes chemical compositions of fabricated MAEs. Obviously, addition of iron to the elastomer matrix increases the shear modulus of the composite material (filler reinforcement). To keep the effective shear modulus of composite materials with two different filling factors, one has to adjust the shear modulus of the elastomer matrix. The matrix should be softer for the material with the higher filler content. Therefore, five different chemical compositions were required. The general approach to chemical synthesis of soft polydimethylsiloxane (PDMS) matrices is described in detail in [62]. Modification of the shear modulus occurred in two ways. For achieving the shear modulus of about 30-50 kPa, it was sufficient to vary the ratio of molar concentrations of vinyl and hydride groups in the initial compound by changing the crosslinker content. For a stiffer material with 120 kPa, the concentration of silicone oil (plasticizer) was further reduced.

As a mold, we employed a flat tissue culture test plate (OrPlate) with 24 wells from Orange Scientific NV/SA (B-1420 Braine-L’Alleud, Belgium). For experiments, samples with a height of approximately 5, 8, and 10 mm were made. The air bubbles were removed using a vacuum desiccator for about 10 min. Finally, the MAE cylinders were precured in the universal oven Memmert UF30 (Memmert GmbH, Schwabach, Germany) at 80 °C for 1 h and then postcured at 60 °C for 24 h with air circulation. The curing was performed in the absence of a magnetic field. Therefore, no alignment of magnetic particles has been achieved and the materials should be random heterogenous and isotropic. Note that, for highly loaded MAEs, in particular with 80 mass% of iron, one could expect the existence of the three-dimensional magnetic–filler network already in the absence of magnetic field [54].

**Figure 1:**
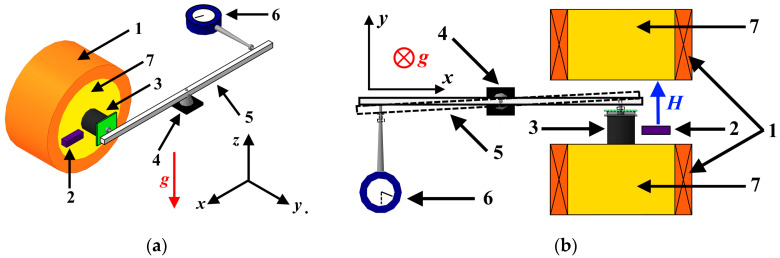
Schematic diagram of the experimental setup for measuring the extensional strain of MAE cylinders: (**a**) three-dimensional view; and (**b**) top view. (1) Electromagnetic coils; (2) Magnetic field sensor; (3) MAE cylinder; (4) pivot point with bearings on a stand; (5) non-magnetic lever; (6) digital indicator with a shaft; and (7) electromagnet poles. The magnetically soft iron yoke is not drawn for clarity of the figures. ***g*** denotes the gravity vector.

Figure 1 shows the schematic view of measurement setup developed at MIREA. A homogeneous magnetic field along the axis of the sample was generated by an electromagnet. The field was directed horizontally. The magnitude of the magnetic field was measured using a Hall sensor-based magnetometer. Direct measurements of the field uniformity showed that it was better than 0.7% over the volume of the sample. A magnetoactive elastomer was adhered at one cylinder base to one of the poles of an electromagnet with a silicone glue. A thin rigid acrylic glass plate was glued to the free base of the cylindrical sample. The glue drops were positioned in the center of the cylinder base and kept as small as possible, to allow for the free deformation of the base circumference. The thickness of the acrylic glass plate was about 100 μm, i.e., much less than the height of the test sample. The area of the plate was about 15 mm × 15 mm and its mass was about 0.03 g. A bronze pin at the end of a non-magnetic (aluminum) lever with a cross-section of 10 mm × 10 mm leaned against the glass plate. The pin and the plate were in permanent contact, thereby avoiding the local deformation of elastomer. The opposite end of the lever rested against the shaft of a digital indicator (model ИЦ 0-12.5 0.001 КЛБ) for the length measurement. An alternative technical solution for measuring the lever deflection could be a LVDT. A digital indicator is a low-cost device in comparison with LVDT. When the magnetic field changed, the sample was deformed. As a result, the lever deviated and affected the shaft of the digital indicator. A similar geometry for measuring magnetostrictive deformations of metals was proposed 95 years ago in [63]. Some preliminary measurements of MS in MAE cylinders have been reported in [64]. The total length of the lever was 43 cm. The digital indicator had a resolution of 1 μm. All measurements were made at room temperature.

The measurements were performed according to the following protocol. The measurement time for one point was 1.5 min. The measurements were carried out in the magnetic field range from 0 to about 400 kA/m with different polarity (positive or negative). This is the maximum field that can be generated by this electromagnet. It corresponds to the magnetic flux density in the air of about 0.5 T. The magnetic field was changed in steps of ≈ 8 kA/m. The initial height of the sample was $h_{0}$. The engineering normal strain was defined as $e={\Delta h/h}_{0}$, where $\Delta h=h-h_{0}$ is the change in the height of the sample.

## 3. Results and Discussion

### 3.1. General Behavior of MAE Strain Loops

**Figure 2:**
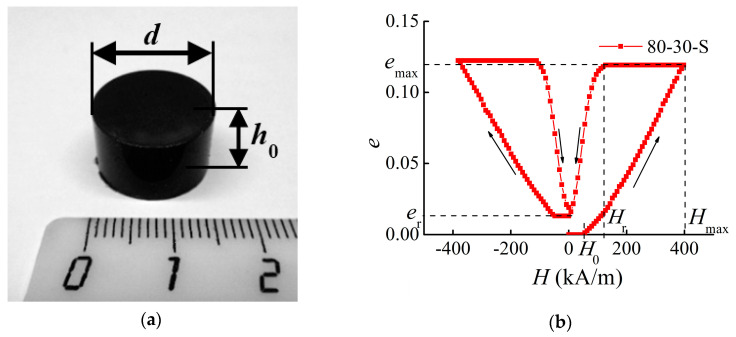
(**a**) Photograph of a sample with dimensions. (**b**) The engineering normal strain $e={\Delta h/h}_{0}$ as a function of the applied magnetic field H. The arrows denote the direction of the field change.

Figure 2a shows a MAE specimen with designated dimensions and Figure 2b presents the measurement results of an exemplary MAE sample and explains the extracted parameters of the specimens. In the initial state, the sample is not deformed and at least 24 h had passed since the last magnetization cycle. When the magnetic field commences to increase from zero, there is initially no deformation (*e* = 0) and a measurable elongation of the sample is observed when the field exceeds a particular threshold field $H_{0}$. $e_{\max}$ denotes the maximum strain in the field $H_{\max}\approx 400$ kA/m, reached for the ascending external field in the first magnetization cycle. $e_{\max}$ is a characteristic of a sample with given dimensions, made of a particular material, subjected to a maximum field. After the maximum field has been reached, the external field is stepwise reduced to zero (first magnetization cycle). In the descending part of the strain hysteresis curve, the engineering strain starts to decrease from a specific value of the external field $H_{r}$. $e_{r}$ is the remanent strain in zero field. Similar parameters can be defined for the following second cycle, where the field direction is reversed. In the second cycle, the magnitude of the magnetic field reaches the value of $\left|-H_{\max}\right|=H_{\max}$, and then it is further reduced to zero. However, the characteristic values differ only slightly from the values in the first cycle, and, therefore, the conclusions made for the described parameters are valid for the second cycle as well.

Table 2 summarizes the results of measurements for all samples with the shear modulus of about 30 kPa. The value of the shear modulus is given in the absence of a magnetic field. 

Table 3 presents the results of measurements for samples with 80 mass% of iron and different effective shear storage moduli. The values of the effective shear storage modulus are given in the absence of a magnetic field.

The following conclusions can be drawn from the analysis of the experimental results. 

### 3.2. Effect of the Aspect Ratio on the Maximum Strain

**Figure 3:**
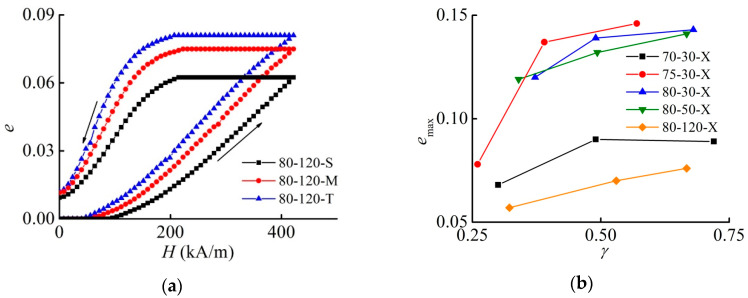
(**a**) Field dependence of the engineering normal strain of MAE samples with 80 mass% of iron and the quiescent shear modulus of 120 kPa for different aspect ratios. (**b**) Dependence of the maximum strain $e_{\max}$ on the aspect ratio γ for different samples.

With an increase in the aspect ratio, the maximum engineering strain increases. Figure 3 illustrates this conclusion. This finding contradicts the results reported in [39], where it was found that the maximum strain decreases with the increasing aspect ratio $\gamma =h_{0}/d$ for the same range of γ. A simple theory developed in [39] predicts the following dependence of the magnetostrictive strain on the sample parameters

(1) $$e=-\frac{\mu_{0}M^{2}}{2E}\gamma (1+\nu )\frac{dN}{d\gamma },$$

where *M* is the magnetization of the sample, *E* is its Young’s modulus, *ν* is the Poisson’s ratio, and *N* is the demagnetizing factor along the cylinder axis. Although the authors did not provide the formula for the demagnetizing factor *N*, it seems that they used the approximate expression of Sato and Ishii [65] for the demagnetizing factor of a cylinder magnetized uniformly along the symmetry axis: $N={\left[\left(4\gamma /\sqrt{\pi }\right)+1\right]}^{-1}$.

**Figure 4:**
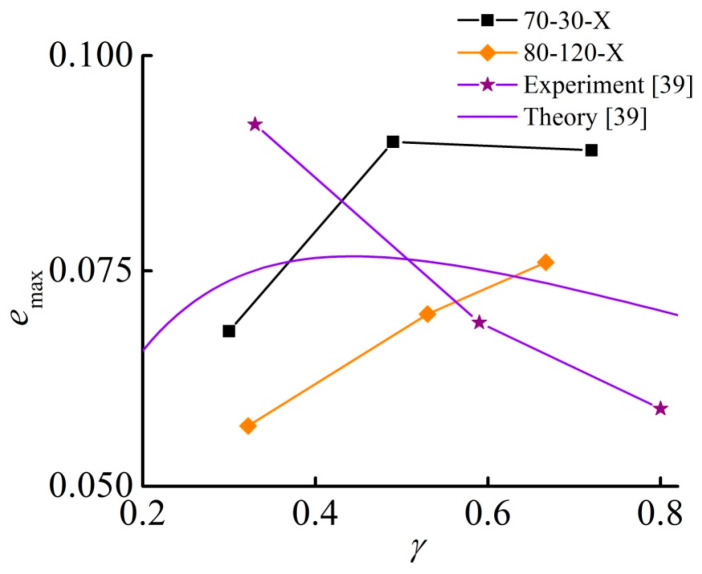
Comparison of experimental and theoretical results obtained in [39] with similar experimental observations in the present work.

Figure 4 compares the experimental and theoretical results of Diguet et al. [39] with our most similar samples as far as the strain values are concerned. The difference is clearly seen: there was no agreement between Equation (1) and the experimental results in [39]. In addition, in the present study, the dependence of the maximum deformation $e_{\max}$ on the aspect ratio γ is qualitatively different from the experimental results of [39], which have been obtained on magnetically saturated samples. Obviously, the approximation of a uniformly magnetized cylinder is not valid for our experimental conditions, because the shape is different from that of a rotational ellipsoid and the magnetic field is relatively low (there is no sign of magnetic saturation in the field dependence of magnetostrictive strain). Moreover, the magnetization *M* may also depend on the aspect ratio γ. However, our results can be qualitatively explained by the influence of the demagnetizing field by assuming that the samples are short and the magnetization in the maximum field does not change much with γ. The derivative dN/dγ is in general negative and N tends to zero with the increase of γ. The product −γ(dN/dγ) can be expected to have a local maximum in its dependence on γ as formula (1) predicts. An indication of the appearance of a local maximum in the dependence of the maximum strain on the aspect ratio can be speculated for the 70-30-X samples. Since only elongations (i.e., positive strains) are observed, according to the existing physical picture, the macroscopic interaction between the magnetization vector and the demagnetizing field dominates over the interaction effect between magnetized particles. It was predicted that a spheroidal sample with isotropic distribution of particles will always elongate along the field if the concentration of particles is high due to steric hindrance between the particles which leads to pronounced non-affinity at local scales [53]. Note that the maximum field in our experiments is about 2.4-fold lower than in [39], but the maximum strain is roughly 1.6-fold higher, which can be explained by the softness of our samples.

### 3.3. Effect of the MAE Shear Modulus on the Maximum Strain

**Figure 5:**
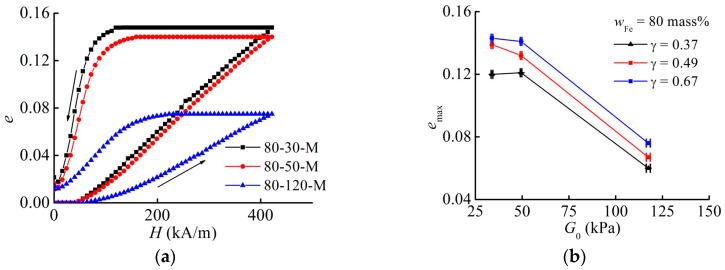
(**a**) Field dependence of the engineering normal strain of MAE samples with 80 mass% of iron and the middle aspect ratio on the shear modulus of the composite material. (**b**) Dependence of the maximum strain $e_{\max}$ on the shear modulus for varied aspect ratio γ.

**Figure 6:**
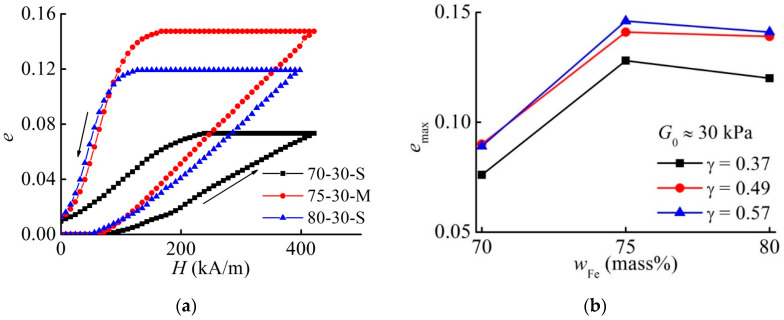
(**a**) Field dependence of the engineering normal strain of MAE samples with the shear modulus of 30 kPa and the aspect ratio of ≈ 0.37. (**b**) Dependence of the maximum strain $e_{\max}$ on the mass fraction of iron particles for different aspect ratios for materials with the same shear modulus of 30 kPa.

With an increase in the shear modulus at the same γ, the maximum relative strain decreases, as demonstrated in Figure 5. This can be explained by a larger mobility of filler particles inside a softer elastomer matrix, leading to the higher magnetostrictive strain. However, this decrease is not inversely proportional to the shear modulus as Formula (1) suggests. In Figure 5b and Figure 6b, the shown values of $e_{\max}$ were obtained by a linear interpolation between the neighboring experimental points in Figure 3b, because the cylinders have slightly different aspect ratios.

### 3.4. Effect of the Magnetic Filler Concentration on the Maximum Strain

Next, we verified the prediction of [39] that there is an optimum volume fraction of 0.27 for the maximum magnetostrictive strain. Such a concentration corresponds to the mass fraction of iron of 75 mass%. Figure 6 demonstrates the experimental results. Figure 6a exemplarily compares three samples with the same effective shear modulus and approximately the same aspect ratio. Indeed, a slightly larger magnetostrictive strain is observed for 75 mass% than for 80 mass% in Figure 6b.

### 3.5. Dependence of the Threshold Field $H_{0}$ on MAE Sample Parameters

**Figure 7:**
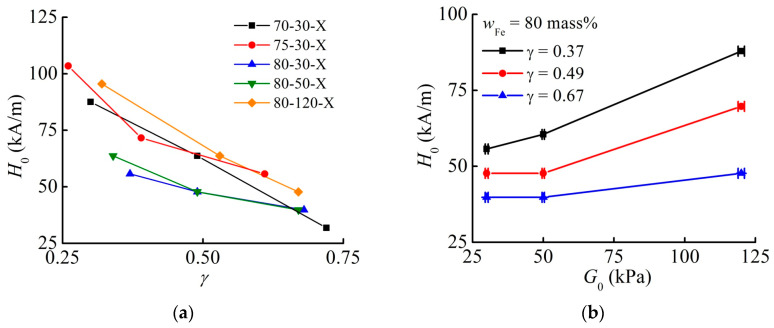
(**a**) Dependence of the threshold field $H_{0}$ on the aspect ratio γ for different MAE samples. (**b**) Dependence of the threshold field $H_{0}$ on the shear modulus $G_{0}$ for the fixed iron content of 80 mass% and varied aspect ratio γ.

Figure 7 is devoted to the dependencies of the threshold field $H_{0}$, in which the deformation starts. In Figure 7b, the shown values of $H_{0}$ were obtained by a linear interpolation between the neighboring experimental points in Figure 7a, because the cylinders have slightly different aspect ratios. The existence of the field $H_{0}$ is related to the force required by the digital indicator to change the numerical value. This nominal force is 1.5 N. In the field $H_{0}$, the magnetic interactions between particles begin to sufficiently prevail over elastic forces trying to restore the initial positions of magnetic particles in the MAE composite, and the resulting mechanical stress [66,67] produces the force capable of moving the digital indicator shaft. Since the internal field inside the MAE material is larger for the larger aspect ratio, $H_{0}$ is decreasing with the growing aspect ratio γ (see Figure 7a) and increases with the increasing shear modulus of the composite materials. $H_{0}$ is not significantly changed for the second magnetization cycle, when the magnetic field changes polarity.

The observed dependence of the threshold field $H_{0}$ on the sample parameters can be rationalized by a recently proposed theoretical framework [67]. There, a formula is presented to predict the pressure acting additionally onto the measuring device of a therein clamped, i.e., undeformed, MAE sample upon applying an external magnetic field. Essentially, this “magnetically-induced” pressure $\Delta p_{mag}$ depends not only on the volume fraction of the magnetizable phase in the sample φ, but also on the actual macroscopic shape of the sample, the microstructural arrangement of the particles, and how the composite-material microstructure couples to the macroscopic form. Explicitly, the $\Delta p_{mag}$ reads:

(2) $$\Delta p_{mag}={\frac{\mu_{0}}{2}\varphi {\bar{M}}^{2}\left(\varphi \frac{\partial f_{macro}}{\partial e}+\frac{\partial f_{micro}}{\partial e}\right)|}_{e=0}.$$

Here, $\mu_{0}=4\pi \cdot 10^{-7}$ H/m is the magnetic permeability of vacuum and $\bar{M}$ denotes the average magnetization of the magnetic inclusions. The parameter $f_{macro}=f_{macro}\left(\gamma \right)$ is a function of the aspect ratio γ. It represents the contribution due to the macroscopic shape of the sample while $f_{macro}$ describes the contribution of the actual microstructure of the filler. The derivatives in Equation (2) are taken in the undeformed state of the samples, e=0. At given $H_{0}$, the average magnetization $\bar{M}$, is obtained via:

(3) $$\bar{M}=L\left[H_{0}+\left(\varphi f_{macro}+f_{micro}-\frac{1}{3}\right)\bar{M}\right],$$

where L(H) represents the magnetization curve of the filling material and 1/3 is the demagnetizing factor of an individual spherical filler particle. Since an external field $H_{0}$, where the sample begins to deform, is rather small, i.e., the threshold field is far from the saturating field strength, we can safely consider a linear magnetization function M=χH with the initial susceptibility χ. In the linear regime, Equation (3) can be analytically solved in a self-consistent manner giving [67]:

(4) $$\bar{M}=\frac{H_{0}}{\frac{1}{\chi }+\frac{1}{3}-\varphi f_{macro}-f_{micro}}.$$

Using Equation (4) in Equation (2), we find the following dimensionless relation of $H_{0}$ to the sample parameters:

(5) $$H_{0\mathrm{norm}}=H_{0}\sqrt{\frac{\mu_{0}}{2\Delta p_{mag}^{*}}}=\frac{\frac{1}{\chi }+\frac{1}{3}-\varphi f_{macro}-f_{micro}}{\sqrt{\varphi^{2}{f^{\prime }}_{macro}+\varphi {f^{\prime }}_{micro}}},$$

where $\Delta p_{mag}^{*}$ is a fixed quantity for all samples as it is purely imposed by the measuring device. $\Delta p_{mag}^{*}$ has the physical meaning of the critical pressure which is necessary for moving the shaft of the digital indicator. 

Note that $f^{\prime }$ in the denominator denotes a partial derivative with respect to the extensional strain in the yet undeformed state e=0. The function $f_{macro}=f_{macro}\left(\gamma \right)$ is related to the demagnetizing factor *N* via $f_{macro}=1/3-N$ [67,68], where *N* for a cylinder with the aspect ratio γ is calculated in [69]. The corresponding derivative ${f^{\prime }}_{macro}$ can be found straightforward assuming material’s incompressibility and uniaxial behavior, which appear reasonable for the presented materials and the setup. The initial susceptibility of the iron-based inclusions was set to χ=131 in agreement with previous works [70,71]. The volume fractions of magnetizable particles in experimental samples are given. Unknown, and practically unattainable, in Equation (5) are the actual microstructural contribution $f_{micro}$ and its derivative ${f^{\prime }}_{micro}$, which describe the coupling of the local composite-material microstructure to the macroscopic deformation (in the onset of deformation). From theoretical analysis [53,72,73], it is known that the both parameters should be in the range (−1; 1). For perfectly isotropic particle distributions, $f_{micro}=0$, whereas chain-like aggregates aligned with an external field exhibit $f_{micro}>0$ and the aggregates aligned perpendicular to an applied field yield $f_{micro}<0$. More difficult are qualified statements concerning ${f^{\prime }}_{micro}$, since the coupling to the macroscopic state is a non-trivial problem and calculation requires additional assumptions such as affinity, restructuring processes, and the role of steric repulsion between neighboring particles.

**Figure 8:**
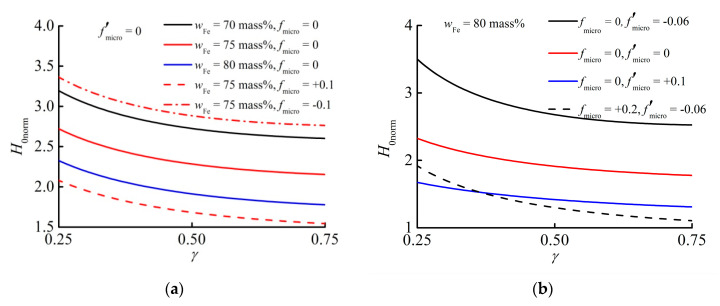
Theoretical dependence of the normalized threshold field $H_{0\mathrm{norm}}$ on the aspect ratio γ. (**a**) The derivative ${f^{\prime }}_{micro}$ =0 is fixed. The red dashed curves show the role of sign of $f_{micro}\neq 0$ at $w_{\mathrm{Fe}}=75\;\mathrm{mass}\%$. Other colors designate varying content of iron particles in samples with identical $f_{micro}=0$. (**b**) $w_{\mathrm{Fe}}=80$ mass% is constant. Solid lines correspond to different ${f^{\prime }}_{micro}$ at fixed $f_{micro}=0$. The dashed curve illustrates the influence of combined positive $f_{micro}$ and negative ${f^{\prime }}_{micro}$ in Equation (5).

In Figure 8, we plot Equation (5) in dependence from possible values of $f_{micro}$ and ${f^{\prime }}_{micro}$ for the three volume fractions of particles in the experimental samples. The trends are quite obvious. First, as also found in experiments (Figure 7a), the threshold magnetic field $H_{0}$ necessary to overcome some fixed barrier (in the theory $\Delta p_{mag}$ correspondingly) reduces with increasing aspect ratio γ, independently of chosen values for $f_{micro}$ and ${f^{\prime }}_{micro}$. Secondly, we notice that an increasing φ may considerably reduce this field $H_{0}$ assuming microstructural parameters to be identical. Nevertheless, the dashed curves in Figure 8a clearly demonstrate that changing $f_{micro}$ to some small positive, or negative, values greatly influences this trend. Accordingly, a slightly more structured particle arrangement with considerably less particles can compensate this effect. An identical trend is also found with respect to the parameter ${f^{\prime }}_{micro}$, as shown in Figure 8b. Thus, positive values of $f_{micro}$ and/or ${f^{\prime }}_{micro}$ may considerably reduce the field $H_{0}$, whereas negative values increase $H_{0}$. However, combining the effect of a slightly negative ${f^{\prime }}_{micro}$ with some positive $f_{micro}$ may also considerably influence the slope of $H_{0}=H_{0}\left(\gamma \right)$. Note that, especially in soft samples, it appears reasonable that the particles may arrange into columnar-like structures along the external field, whereas in stiffer samples such effects should be suppressed. Hence, softer samples should display lower $H_{0}$ than stiffer samples.

Comparing these theoretically found trends to the experimental data plotted in Figure 7, we find some reasonable agreement. All curves display an identical decrease with increasing γ where the steepest slopes are found at rather low γ. Although one may expect that samples 70-30-X and 75-30-X should only differ in their particle loadings, and thus the curve for 75-30-X should be shifted clearly below curve 70-30-X, some differences in the microstructure, or its coupling to the macro-state in the process of cross-linking, during preparation can easily compensate for such a shift. In contrast, the curve 80-30-X is clearly found below the curve 75-30-X. Concerning the stiffness variation among the experimental samples 80-30-X, 80-50-X, and 80-120-X, another theoretical speculation is confirmed. Clearly, the stiffest sample displays the highest field $H_{0}$, suggesting that beneficial rearrangements into columnar structures are strongly suppressed, in contrast to the two softer samples.

This effect of possibly suppressed rearrangements and, thus, increasing $H_{0}$ for stiffer samples may be directly identified from the plot in Figure 7b, where the threshold field $H_{0}$ is drawn against an increasing shear storage modulus $G_{0}$ of the sample.

### 3.6. Dependence of the Field $H_{r}$ and the Remanent Strain $e_{r}$ on Parameters of MAE Samples

We attribute the existence of the field *H*_r_, where the deformation commences to decline for the descending part of the magnetostriction curve in the first magnetization cycle to the same reason as for $H_{0}$. A certain change in the magnetostrictive stress is required to alter the value of the digital indicator. As $H_{0}$, *H*_r_ increases with the increasing shear modulus at constant filler content. For the constant shear modulus of the composite material, *H*_r_ decreases with the increasing content of filler particles. The last two observations can be explained that *H*_r_ follows the shear modulus of the elastomeric matrix. At constant filler concentration and shear modulus values of the composite material (i.e., the shear modulus of the elastomer matrix is constant), *H*_r_ seems to be practically independent of the aspect ratio γ.

No clear dependence of the remanent strain *e*_r_ ≈ 0.01 on γ, filler content, and the shear modulus of the composite material has been observed. 

### 3.7. Magnetostriction of Isotropic Versus Anisotropic MAE

**Figure 9:**
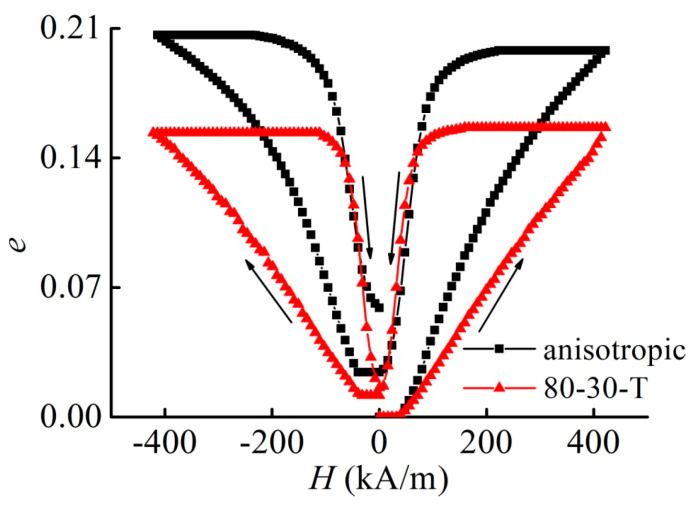
Comparison of the magnetostrictive strain of an anisotropic sample with 80 mass% of iron with the corresponding isotropic sample showing the largest magnetostrictive strain.

Finally, we fabricated for comparison an anisotropic sample with 80 mass% of Fe, where the particles have been pre-aligned along the cylinder axis by crosslinking in a constant magnetic field of 80 mT. Figure 9 compares the magnetostrictive strain of such sample with the similar isotropic MAE cylinder. It is observed that the magnetostrictive strain has been increased by approximately 27%. The higher strain in the anisotropic sample in comparison with the isotropic specimen indirectly confirms the higher impact of the anisotropic magnetic (filler) microstructures predicted theoretically and described by the parameter $f_{micro}$. $f_{micro}$ is positive for the chain-like structures aligned along the magnetic field lines (see the discussion above). For quantifying this effect, more detailed investigation is needed, which is outside of the scope of the present paper.

## 4. Conclusions

The main goal of the present paper is a comprehensive investigation of magnetodeformations in soft magnetoactive elastomers realized in homogeneous magnetic fields. The focus is on the impact of the macroscopic shape of the samples as well as their magneto-mechanical properties such as the shear modulus and magnetic filler content on the magnetostrictive response and its dependence on external ascending–descending magnetic fields.

For this aim, a simple experimental setup was designed for measuring large strains realized in soft MAEs with the shear modulus as low as 30 kPa and a series of MAE cylindrical samples were synthesized. MAEs, used in this study, were based on PDMS matrices and carbonyl iron magnetic microparticles with the average diameter of 4.5 µm. Sets of the samples differing by the volume fraction of the magnetic filler (0.22, 0.27 and 0.33) and the shear modulus (about 30, 50, and 120 kPa) were fabricated to clarify the role of MAE composition in its magnetostrictive behavior. Furthermore, the cylindrical samples with the aspect ratios varying in the range 0.26–0.68 were prepared for different MAE compositions to evaluate the shape contribution into magnetically induced strain of the samples. It was anticipated that it is the restructuring of the magnetic filler that is responsible for material macro-deformations in external magnetic fields. At a fixed value of the magnetic field, this restructuring depends on the interplay of the sample shape and material composition, namely the compliance of the polymer matrix and distribution of the magnetic particles. Thus, extensive variation of the above-mentioned parameters allowed understanding their relative contributions and to shed light into the underlying mechanism of MS in soft MAEs.

First, it should be noted that the fabricated soft MAE cylinders demonstrated huge elongations up to 0.21 in the moderate magnetic field of 400 kA/m applied along the cylinder axis. The obtained values of MS can be considered as a record. Second, the magnetostrictive behavior of all the samples in ascending–descending magnetic fields was characterized by a significant hysteresis, which is typical for magneto-mechanical properties of soft MAEs. Third, there was a pronounced dependence of MS elongation on the macro- and micro-characteristics of MAE samples. 

In contrast to some results reported in the literature, MAE elongations in the maximum magnetic field grew with an increase of the aspect ratio γ of the cylinders. It should be noted that this result was obtained for low values of γ, i.e., the MAE cylinders in this study were rather short, and further measurements for longer samples seem to be very promising for getting a general picture of the relative influence of the form factor and magnetic microstructure on MS in MAEs.

The effect of the material composition on MS seems to be defined by the extent of magnetic filler rearrangements in magnetic fields. MAE strains drop with an increase of the sample shear modulus, which can be explained by enhanced restrictions on particle restructuring for elastically rigid composites. An existence of an optimum volume fraction of magnetic particles for realization of maximum strains, which was observed experimentally, can also be caused by suppressed particle restructuring at a higher filler content due to a tight packaging of the particles. 

The application of a theoretical framework, which allows one to separate the contributions to the magnetic stress due to the macroscopic shape of the sample and the internal microstructure of the material, makes it possible to rationalize the experimental results for the dependence of the threshold magnetic field, in which the registration of the sample elongation begins, on the macro- and micro-parameters of MAEs. Qualitative agreement between the experimental data and theoretical predictions shows the prospect of this integrated approach to evaluate quantitatively the impact of the internal MAE microstructure on magnetically induced stress. In this respect, it seems to be very promising to perform extensive comparative studies of MS in isotropic and anisotropic samples with pre-aligned magnetic filler aggregates. Preliminary results reported in the present paper demonstrate a considerable enhancement of MS in structured composites.

## Figures and Tables

**Table 1:** Proportions of individual components for fabrication of the MAE samples in mass percent.

Samples (Material)	CIP	AK 10	VS 100000	MV 2000	Cross-linker 210	Pt Catalyst	Inhibitor	Modifier
70-30-X	69.769	19.914	8.448	1.509	0.179	0.010	0.050	0.030
75-30-X	74.801	16.606	7.045	1.258	0.141	0.083	0.042	0.025
80-30-X	79.835	13.292	5.639	1.007	0.106	0.067	0.033	0.020
80-50-X	79.822	13.290	5.638	1.007	0.123	0.066	0.033	0.020
80-120-X	79.806	11.386	7.246	1.294	0.143	0.067	0.033	0.026

**Table 2:** Measurement results for MAE samples with the shear modulus of about 30 kPa.

Sample	70-30-S	70-30-M	70-30-T	75-30-S	75-30-M	75-30-T	80-30-S	80-30-M	80-30-T
*w*_Fe_, mass%	70	70	70	75	75	75	80	80	80
*d*, mm	14.9	15.2	14.8	14.8	14.9	14.5	14.5	14.7	14.7
*h*_0_, mm	4.4	7.4	10.7	3.8	5.8	8.2	5.4	7.2	10.0
*γ* = *h*_0_*/d*	0.30	0.49	0.72	0.26	0.39	0.57	0.37	0.49	0.68
*H*_0_, kA/m	88	64	32	103	72	56	56	48	40
*e*_max_	0.068	0.09	0.089	0.078	0.137	0.146	0.12	0.139	0.143
*H*_r_*,* kA/m	231	239	271	127	159	179	119	111	151
*e*_r_	0.010	0.013	0.010	0.012	0.014	0.013	0.013	0.014	0.0117

**Table 3:** Measurement results for MAE cylinders with 80 mass% of carbonyl iron.

Sample	80-30-S	80-30-M	80-30-T	80-50-S	80-50-M	80-50-T	80-120-S	80-120-M	80-120-T
*w_Fe_*, mass%	80	80	80	80	80	80	80	80	80
*d*, mm	14.5	14.7	14.7	14.7	14.8	14.7	15.2	14.9	15.0
*h*_0_, mm	5.4	7.2	10.0	5.0	7.3	9.8	4.9	7.9	10.0
*γ = h*_0_ */d*	0.37	0.49	0.68	0.34	0.49	0.67	0.32	0.53	0.67
*H*_0_, kA/m	56	48	40	64	48	40	95	64	48
*e*_max_	0.120	0.139	0.143	0.119	0.132	0.141	0.057	0.070	0.076
*H*_r_*,* kA/m	119	111	151	159	151	159	207	215	199
*e*_r_	0.013	0.014	0.0117	0.011	0.011	0.011	0.009	0.012	0.012
